# Double quantum criticality in superconducting tin arrays-graphene hybrid

**DOI:** 10.1038/s41467-018-04606-w

**Published:** 2018-06-04

**Authors:** Yinbo Sun, Hong Xiao, Miao Zhang, Zhongying Xue, Yongfeng Mei, Xiaoming Xie, Tao Hu, Zengfeng Di, Xi Wang

**Affiliations:** 1 State Key Laboratory of Functional Materials for Informatics, Shanghai Institute of Microsystem and Information Technology, Chinese Academy of Sciences, 865 Changning Road, Shanghai, 200050 China; 2 University of Chinese Academy of Sciences, Beijing, 100049 China; 3 Center for High Pressure Science and Technology Advanced Research, Beijing, 100094 China; 4 Department of Materials Science, Fudan University, Shanghai, 200433 People’s Republic of China; 5 CAS Center for Excellence in Superconducting Electronics (CENSE), Shanghai, 200050 China

## Abstract

Two magnetic-field-induced quantum critical behaviors were recently discovered in two dimensional electron gas (2DEG) at LaTiO_3_/SrTiO_3_ interface and interpreted by disordered superconducting puddles coupled through 2DEG. In this scenario, the 2DEG is proposed to undergo a spontaneous phase separation and breaks up into locally superconducting puddles in a metallic matrix. However, as the inhomogeneous superconducting 2DEG is only illative, this proposal still lacks the direct experimental demonstration. Here, we artificially construct superconducting puddles-2DEG hybrid system by depositing tin nanoislands array on single crystalline monolayer graphene, where the two quantum critical behaviors are reproduced. Through the finite-size scaling analysis on magnetoresistivity, we show that the two quantum critical behaviors result from the intra-island and inter-island phase coherence, respectively, which are further illustrated by the phase diagram. This work provides a platform to study superconducting quantum phase transitions in a 2D system and helps to integrate superconducting devices into semiconductor technology.

## Abstract (web summary)

Two quantum critical behaviors appear in a two dimensional electron gas (2DEG) but its origin remains to be attested. Here, Sun et al. construct superconducting puddles-2DEG hybrid system by depositing tin nano-islands array on monolayer graphene where the two quantum critical behaviors are reproduced, suggesting the formation of inhomogeneous superconducting 2DEG.

## Introduction

Novel quantum phenomena, like high temperature superconductivity^1–3^, Bose metallic state^4,5^, and quantum griffiths^6^ phase were always observed in 2D superconducting material and their properties were believed to be controlled by a continuous quantum phase transition (QPT) at zero temperature. The QPT occurs at zero temperature as a result of the change of the Hamiltonian under the non-thermal parameters, like disorder, the magnetic field or the doping^7^. The quantum criticality arising from a continuous QPT can govern the phase diagram up to very high temperature and be described by the theory of finite-size scaling (FSS). The conventional perpendicular magnetic field tuned QPT is always single in the amorphous or granular 2D superconductors^8–13^. While recent experiments instead showed superconductor to insulator phase transitions (SIT) tuned by the magnetic field corresponding to two QPTs at LaTiO_3_/SrTiO_3_ interface^14^. Previous theoretical studies suggest that the QPT, even for multiple QPTs, may be induced by inhomogeneities of superconductivity spontaneously developed near the transition in two dimensions^15,16^. Thus, the two QPTs emerged at LaTiO_3_/SrTiO_3_ interface were also assumed to be related to the inhomogeneous superconductivity, in which one QPT corresponded to the local ordering of superconducting puddles formed by high-mobility carriers (HMCs) and the other QPT was due to the coherence between superconducting puddles coupled by 2DEG^14^. However, since the existence of superconducting puddles and 2DEG at LaTiO_3_/SrTiO_3_ interface could not be visually observed, the physical mechanism underlying multiple QPTs in 2D superconductors is still under debate. To elucidate the origin of the observed multiple QPTs, a visualized platform to reproduce the system consisting of the illative superconducting puddles-2DEG system at LaTiO_3_/SrTiO_3_ interface is essentially demanded.

**Fig. 1:**
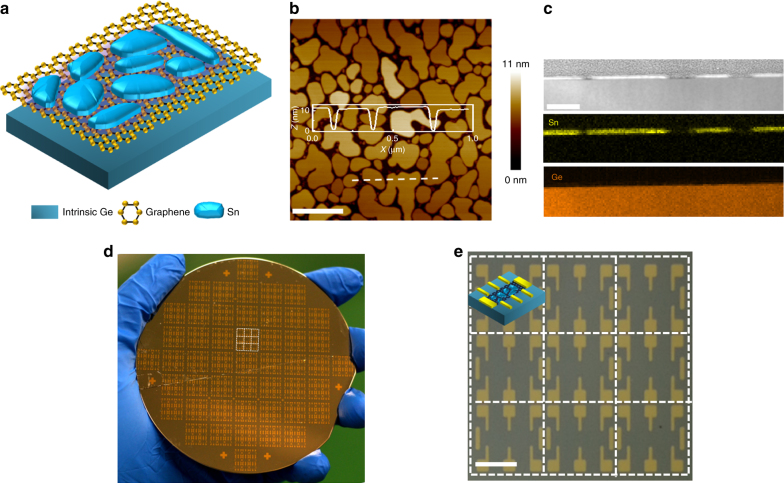
Graphene–tin nanoislands hybrid system on 4 inch intrinsic germanium wafer. **a** Sketch illustration of the designed system consisting of tin nanoislands-decorated single crystalline monolayer graphene. **b** Atomic force microscopy (AFM) image of discontinuous tin islands with a uniform thickness of 10 nm self-assembled on single crystalline graphene. The scale bar is 500 nm. Inset: The depth profile along the white dash line in Fig. 1b. **c** STEM image of the cross sectional 10 nm-thick discontinuous tin-nanoislands on the graphene, and the corresponding STEM-EDS mapping images revealing the distributions of Sn and Ge. The scale bar is 100 nm. **d** Photograph of the test device matrixes fabricated on 4 inch intrinsic germanium wafer. **e** Magnified view from **d** shows nine individual superconducting devices assembled into one array. The scale bar is 2 mm

Here, we artificially fabricated the inhomogeneous 2D superconductor consisting of superconducting tin nanoislands and single crystalline monolayer graphene, in which an array of disordered superconducting tin nanoislands can visually mimic the suspected superconducting puddles and single crystalline monolayer graphene provides two dimensional electron gas (2DEG), as schematically illustrated in Fig. 1a. The obtained graphene-tin nanoislands array hybrid exhibits the ideal 2D superconductivity at low temperature. As increasing the perpendicular magnetic fields, the graphene-tin nanoislands array hybrid is driven from the 2D superconductor to the weakly localizing metal. Along with the transition process, we observe two quantum criticalities, which correspond to the intra-tin nanoisland superconductivity and the inter-tin nanoisland superconductivity, respectively. The constructed *H*–*T* phase diagram further verifies the presence of two QPTs in graphene-tin nanoislands array hybrid.

## Results

### Fabrication of inhomogeneous 2D superconductor

Four inch intrinsic Ge (110) wafer was chosen as the starting platform for the synthesis of single crystalline monolayer graphene^17,18^ (Supplementary Figure 1). By using the stencil mask, the standard hall bar device matrixes were subsequently fabricated on graphene wafer. Then, 10 nm-thick elemental superconductor tin was deposited on 4 inch single-crystalline graphene wafer by electron beam evaporation. Due to the poor wettability of graphene and the low melting point of tin^19,20^, an array of self-assembled irregular tin nanoislands with lateral size of ~150 nm and interval of ~40 nm was formed to build graphene-tin nanoislands hybrid system (Fig. 1b, Supplementary Figures 2, 3). The single crystalline graphene provides a strict 2DEG platform, which is rather inert in the environment^21^ and has no grain boundary intervalley carrier-scattering effect^22,23^, while an array of disordered superconducting tin islands visually reproduces the hypothesized superconducting puddles^14^. Both cross sectional scanning transmission electron microscopy (STEM) and STEM-energy dispersive x-ray spectroscopy (STEM-EDS) also suggest that tin nanoislands are discontinuously formed on graphene/Ge (110) substrate (Fig. 1c). To avoid the possible degradation of tin nanoislands due to oxidation, the device array (Fig. 1e) was immediately diced from the whole wafer (Fig. 1d) and transferred into the dilution refrigerator for four-terminal transport measurements.

### Two dimensional superconductivity

**Fig. 2:**
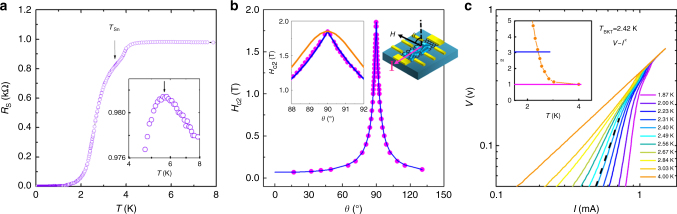
Two dimensional superconductivity of graphene–tin nanoislands array. **a** The four-terminal sheet resistance (*R*_s_) versus temperature (*T*). The black arrow indicates the critical temperature of bulk tin *T*_Sn _= 3.7 K. Inset: *R*_s_-*T* curve on the semi-logarithmic scale from 4 K to 8 K. The *R*_s_ peak hints the superconducting fluctuation. **b** The upper critical fields *H*_c2_ as a function of the angle *θ*. *θ* is the angle between the magnetic field and the surface normal direction of the device with current flowing always in plane, as sketched in the right inset. The blue solid line and the orange solid line correspond to the theoretical fitting of the *H*_c2_(*θ*) using the 2D Tinkham formula $(H_{{\mathrm{c}}2}(\theta )\sin \theta /H_{{\mathrm{c}}2}^\parallel )^2 + |H_{{\mathrm{c}}2}(\theta )\cos \theta /H_{{\mathrm{c}}2}^ \bot | = 1$ and the 3D anisotropic mass model$H_{{\mathrm{c}}2}(\theta ) = H_{{\mathrm{c}}2}^\parallel /(\sin ^2(\theta ) + \gamma ^2\cos ^2(\theta ))^{1/2}$with $\gamma = H_{{\mathrm{c}}2}^\parallel /H_{{\mathrm{c}}2}^ \bot$, respectively. $H_{{\mathrm{c}}2}^\parallel$ and $H_{{\mathrm{c}}2}^ \bot$ represent the upper critical field parallel and perpendicular to the surface of the device, respectively. **c** Voltage–current (*V*–*I*) curves on logarithmic scale at various temperatures. The black dashed line represents *V* = *I*^3^. Inset shows the extracted power-law fitting exponent *α* as a function of the temperature. The Berezinskii-Kosterlitz-Thouless (BKT) temperature *T*_BKT _= 2.42 K is defined by *α* = 3

As cooling down from 8 K, the sheet resistance (*R*_s_) of the device array at zero magnetic field firstly exhibits a semiconductor-like resistivity behavior (d*R*/d*T*<0) where the sheet resistance increases as the temperature decreases (inset in Fig. 2a), then undergoes two-step superconducting transition process, as demonstrated in Fig. 2a. It is worth noting that the intrinsic Ge (110) substrate becomes totally insulating at 10 K (Supplementary Figure 4), so the shunt effect from the substrate can be securely avoided at the temperature below 10 K. The semiconductor-like resistivity behavior can be understood in terms of a weak localization behavior proposed in 2D metals^19^ which is consistent with the previous observation^24^. The first abrupt change in the resistance slop occurs at around *T*_Sn_ = 3.7 K, which is attributed to the superconducting transition of tin nanoislands as observed previously^19^. While the superconducting fluctuation effect of tin islands emerges far above *T*_Sn_, which drops the weak localization resistance and induces a small peak at 5.6 K, as observed in inset of Fig. 2a. When further cooling down, those superconducting tin nanoislands can couple to each other through the 2DEG in graphene as a Josephson junction arrays^19,25^, where the competition between the charging energy *E*_c_ and the Josephson coupling energy *E*_j_ of the superconducting islands is responsible for the global superconductivity^19,26–28^. Note that the coupling effect from the 2DEG in graphene is verified by the comparison experiment (Supplementary Figure 5) in which the device becomes insulating after the removal of graphene between adjacent tin nanoislands by oxygen plasma etching. Thus, the second abrupt change in the resistance slop at lower temperature can be understood as the onset of the global superconducting phase coherence aided by the 2DEG arising from single crystalline monolayer graphene.

The upper critical field (*H*_c2_) of the global superconductivity as a function of *θ* at 2 K is demonstrated in Fig. 2b. Here, *H*_c2_ is defined as the magnetic field where the sheet resistance becomes 50% of the normal state resistance as shown in Supplementary Figure 6. It is found that the sharp peak of *H*_c2_ observed at *θ* = 90^°^ can be well fitted by the 2D Tinkham model (see the blue line in the left inset of Fig. 2b), but deviates from the 3D anisotropic mass model (orange line in the left inset of Fig. 2b), indicating the behavior of 2D superconductivity. Such a *θ* dependence of *H*_c2_ behavior was also observed in other 2D superconducting thin films, like ZrNCl-EDLT^4^, SrTiO_3_-EDLT^29^, niobium-doped SrTiO_3_ thin film^30^. Figure 2c demonstrates the voltage–current (*V*–*I*) curves of graphene-tin nanoislands array from 1.87 to 4 K in the log-log scale. A power-law dependence of $V \propto I^\alpha$ behavior is observed at each temperature and the extracted exponent *α* increases monotonically with decreasing temperature (inset in Fig. 2c). Since the additional thermometer suggests the temperature variation is less than 0.07 K during the *V*–*I* measurement (Supplementary Figures 7-10), the observed power-law *V*–*I* behavior is not due to the effect of Joule heating, and corresponds to the Berezinskii-Kosterlitz-Thouless (BKT) type transition in 2D superconductor^6,31^. The BKT type transition is usually utilized to interpret the unbound vortex–antivortex pairing into bound vortex–antivortex process at low temperature in 2D superconductor, and *V*–*I* curves always meet *V* = *I*^3^ at the BKT transition temperature (*T*_BKT_)^5,6,31^. Here the *T*_BKT_ equals to 2.42 K at *α* = 3, as indicated by the blue solid line in the inset of Fig. 2c, which is consistent with the reported BKT temperature of tin islands coupled by the exfoliated graphene flake^20^.

### Two SITs observed in the graphene-tin nanoislands array

**Fig. 3:**
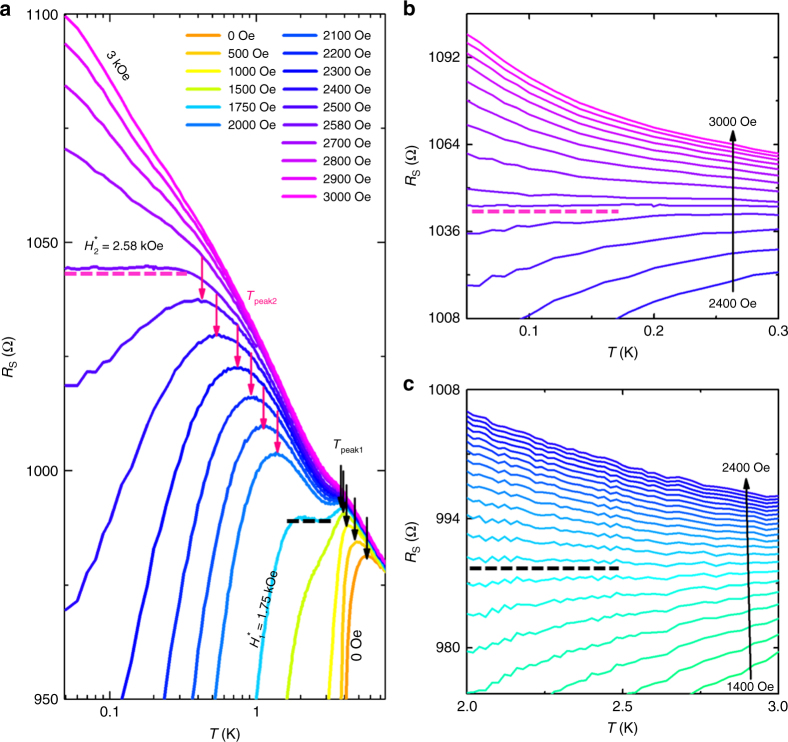
Two QPTs observed in graphene–tin nanoislands array. **a** The sheet resistance *R*_s_ as a function of temperature *T* for different magnetic fields *H* from 0 to 3 kOe. The black and pink arrows indicate the *R*_s_ peaks. **b**, **c** The detailed data collected from the low temperature region (0.05~0.30 K) and high temperature region (2.0~3.0 K) with the magnetic fields varying from 2.4 to 3 kOe and from 1.4 to 2.4 kOe, respectively. The black and pink dashed lines indicate the critical fields *H*_1_* = 1.75 kOe and *H*_2_* = 2.58 kOe, respectively, where the *R*_s_ values are independent of the temperatures

The measurements conducted above confirm that graphene-tin nanoislands array system behaves as a true 2D superconductor since the feature characteristics of 2D superconductivity are obviously observed. The superconductivity in 2D system can be easily tuned into a weakly localizing metal state by the perpendicular magnetic field^6,9,13,14^. Figure 3a (Supplementary Figure 11) demonstrates the *R*_s_(*T*) curves at 0 < *H* < 3 kOe, where the *R*(*T*) curve at each magnetic field is separated by the superconducting (d*R/*d*T* > 0) and weakly localizing metallic (d*R/*d*T* < 0) regions at certain temperature *T*_peak_ (marked with arrows). As the magnetic field increases, the *T*_peak_ shifts to a lower temperature and ends at the *T*-independent resistance region as previously observed in the LaSr_2−*x*_Cu_*x*_O_4_ thin film^1^. The *T*-independent resistance region is indicated by the critical line (the horizontal lines), which is the signature of the continuous QPT. In Fig. 3a, the two critical lines accompanied by the two *T*_peak_ behaviors (pink and black arrows) suggest the presence of two distinct continuous QPTs.

To clarify the nature of two QPTs, we performed the detailed measurements nearby the critical lines as shown in Fig. 3b, c, respectively. It reveals there exist two critical regions where the sign of d*R*/d*T* changes as the magnetic field varies and d*R*/d*T* can approach to zero for certain magnetic field. For high temperature critical region (HTCR), the critical resistance *R*_c_ and the critical magnetic field H^*^ are *R*_c1_ = 988.4 Ω and *H*_1_^*^ = 1.75 kOe, respectively. While, *R*_c2_ = 1044.4 Ω and *H*_2_^*^ = 2.58 kOe are obtained in low temperature critical region (LTCR). It is rather interesting that both critical resistances *R*_c1 _= 988.4 Ω and *R*_c2_ = 1044.4 Ω are significantly lower than the quantum resistance for Cooper pairs *R*_Q _= *h*/4*e*^2^=6.45 kΩ^32^. The early experiments on InO_*x*_ film showed that the disorder of the system had significant influence on the critical resistance^13^. For the strong disordered materials, the critical resistance approaches *R*_Q_ as predicted by the dirty boson model, while the weak disorder system undergoes the QPT from the superconducting state to an metallic phase with *R*_c_ < *R*_Q_^13^. Thus, our graphene-tin nanoislands array system (*R*_c1_/*R*_Q_ = 0.15 & *R*_c2_/*R*_Q_ = 0.16) agrees with the weak disorder picture^13^ due to the weak electronic disorder of single crystalline monolayer graphene.

### Two QPTs identified by the FSS

**Fig. 4:**
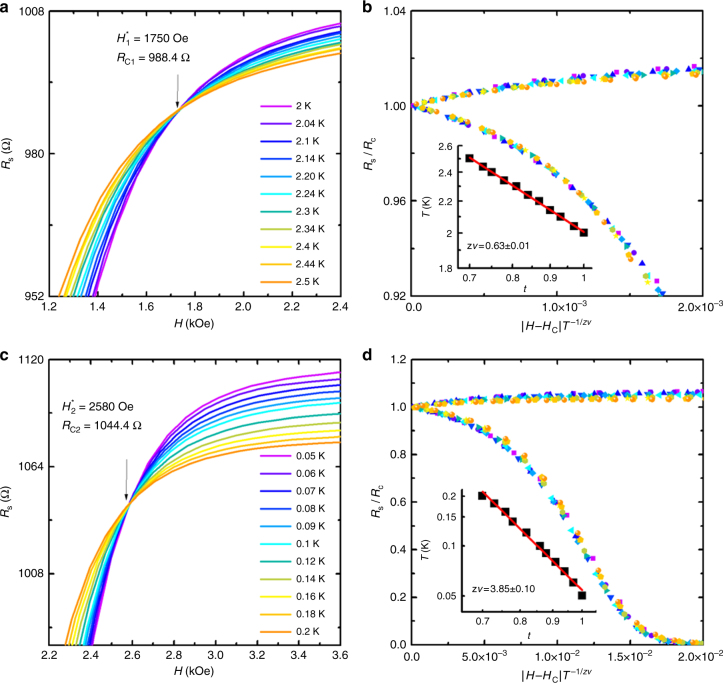
Scaling behavior of the superconductor-insulator quantum phase transition in graphene–tin nanoislands array. **a** Sheet resistance *R*_s_ as a function of magnetic field *H* for different temperatures from 2 to 2.5 K. The black arrow indicates the critical magnetic field and the critical sheet resistance. **b** Finite-size scaling analysis of QPT by utilizing the same data extracted from **a**. Inset: the temperature as a function of the scaling parameter *t*(*T*) on a log-log scale (see the main text for definition of *t*). The power-law fitting determines *zv* = 0.63 ± 0.01. **c** Sheet resistance *R*_s_ as a function of magnetic field *H* for different temperatures from 0.05 to 0.2 K. **d** Finite-size scaling analysis of QPT utilizing the same data extracted from **c**. Inset: the temperature as a function of the scaling parameter *t* on a log-log scale. The power-law fitting determines *zv* = 3.85 ± 0.10

The continuous QPT in Fig. 3 can be further confirmed by the FSS analysis^7^. The FSS states that the *R*_s_(*H*) near the continuous QPT obeys the relationship $R_{\mathrm{s}}(H,T)/R_{\mathrm{c}} = F(|H - H_{\mathrm{c}}|T^{ - 1/zv})$, where *H* is the magnetic field, *H*_c_ is the critical field, *R*_c_ is critical resistance and *F* is an arbitrary function with *F*(0) = 1^32^. The parameter *v* is the correlation length exponent, *z* is the dynamical scaling exponent and $\delta = |H - H_{\mathrm{c}}|$ is the absolute value of distance from the transition, which determine the spatial correlation length *ξ* and the temporal correlation length *ξ*_*τ*_ in *ξ* ~ *δ*^−*v*^ and *ξ*_*τ*_ ~ *ξ*^*z*^ in the vicinity of the continuous QPT. We thus performed the FFS analysis and re-plotted the same sheet resistance data displayed in Fig. 3 as a function of *H* in Fig. 4a, c. It is observed that two sets of *R*_s_(*H*) curves exactly converge at the crossing points (*R*_c1_, *H*_1_^*^) and (*R*_c2_, *H*_2_^*^), respectively (Supplementary Figure 12). The values of crossing points, i.e., *R*_c1_ = 988.4 Ω, *H*_1_^*^ = 1750 Oe, *R*_c2 _= 1044.4 Ω and *H*_2_^*^ = 2580 Oe are consistent with the critical values obtained in Fig. 3. For both HTCR and LTCR, the *R*_s_ decreases at *H* < *H*_c_^*^ while increases at *H* > *H*_c_^*^ when the temperature decreases, which reveals the critical crossing points are temperature independent and separate different quantum ground states.

For HTCR, to yield the best collapse, the method described in ref.^11^ was utilized to obtain the appropriate exponent product *zv* (Supplementary Figures 13, 14). Each magnetoresistivity isotherms curve in Fig. 4a was re-plotted in the form $R_{\mathrm{s}}(H,T)/R_{\mathrm{c}} = F(|H - H_{\mathrm{c}}|,T)$ and multiplied by the factor *t* to acquire the best collapse into the lowest temperature curve with the *t*(*T*_0_) = 1 at the lowest temperature *T*_0_. The temperature dependence of the parameter *t* is the formula: *t* = (*T*/*T*_0_)^−1/*zv*^. The product *zv* is determined by plotting *t* dependence of *T* on a log-log scale with the straight line’s slope equaling to the −*zv*. The *T* versus *t* plot reveals the exponent product value *zv* = 0.63 (Fig. 4b inset). Using this *zv* value, the measured *R*–*H* curves (Fig. 4a) can be scaled into one single curve as shown in the Fig. 4b with respect to the single scaling variable $|H - H_{\mathrm{c}}|T^{ - 1/zv}$. *zv* = 0.63 is consistent with *zv* = 2/3 observed in QPT induced by perpendicular magnetic field in 2D superconductors, like LaTiO_3_/SrTiO_3_ interface^14^, amorphous Nb_0.__15_Si_0.__85_^12^, amorphous Bi^10^ and ultrathin high *T*_c_ superconductor^1^. The dynamical scaling exponent *z* usually equals to 1 in the consequence of long range Coulomb interaction between charges^32,33^ as observed in ultrathin Bi film^11^, amorphous MoGe^9^, unless the particular case in ^4^He porous media with short range Coulomb interactions^34,35^. Hence we can obtain correlation length exponent *v* = 0.63 approximately to 2/3, if takes *z* = 1. Actually, the continuous QPT in 2D superconducting system can be described by the (2+1)D XY model^7^. The *v* = 2/3 is expected in clean (2 + 1)D XY regime^36^, which frequently describes the QPT induced by magnetic field in metallic superconducting films, such as in the amorphous Bi film^10,11^ or ultrathin Be film^37^. Thus the critical behavior in HTCR (*zv* = 0.63) reflects that the QPT in metallic tin nanoislands belongs to the clean (2 + 1)D XY model. In the clean (2 + 1)D XY model, the external magnetic field causes a frustration in the phase coupling^36^, suggesting that the QPT is driven by phase fluctuations at intra-tin nanoislands. In the superconducting LaTiO_3_/SrTiO_3_ interface, the average size of assumed superconducting puddle *L*_d_ is determined by the critical magnetic field as described in $L_{\mathrm {d}}{\mathrm{\sim }}(\Phi _0/H_1^ \ast )^{1/2}$, where *Φ*_0_ is the quantum flux^14,38^. We thus obtain *L*_*d*_ = 118 nm according to$H_1^ \ast = 1750\,{\mathrm{Oe}}$, which is the same order of the typical size of tin nanoislands deposited in our case as shown in Fig. 1 (Supplementary Figure 3).

The magnetoresistivity curves in LTCR (Fig. 4c) can be scaled into one single curve as in Fig. 4d and we can determine *zv* = 3.85, which suggests that *v* = 3.85 and *z* = 1 as discussed above. The exponent product $v \ge 1$ is also observed in the disordered system such as LaTiO_3_/SrTiO_3_ interface^14^, amorphous InO_x_^13^ and amorphous MoGe^9^, which is the consequence of the QPT in 2D dirty regimes^14,39^. As shown in Fig. 1b, the tin islands with irregular shapes are randomly distributed on single crystalline monolayer graphene. The random electric potentials of the tin nanoislands can contribute to the disorder degree in our hybrid system, which induces the macroscopic inhomogeneous superconducting order parameter^40^. Thus, the exponent *v* = 3.85 is related to the QPT of 2D dirty regimes and attributed to the QPT of inter-tin nanoislands superconductivity.

It is worth noting that the electrostatic gating can also induce the QPT in 2D superconductor by changing the carrier density^2,41^. However, the pervious study on the electrostatically gated graphene-tin nanoislands hybrid system only exhibits single QPT^19^, which is different from two QPTs induced by the magnetic field in our work. The possible reason is that the electrostatic gating can alter the Fermi level of graphene and the consequent carrier density^42^, but can not tune the carrier density of metallic tin nanoislands^40^, therefore, only single QPT emerges corresponding to the inter-tin islands physics. As the magnetic field can induce the vortex matter in both the intra-tin and inter-tin nanoisland superconductors, two QPTs can be observed in the graphene-metallic tin nanoislands hybrid system accordingly, other than single QPT emerging during the electrostatic gating.

## Discussion

**Fig. 5:**
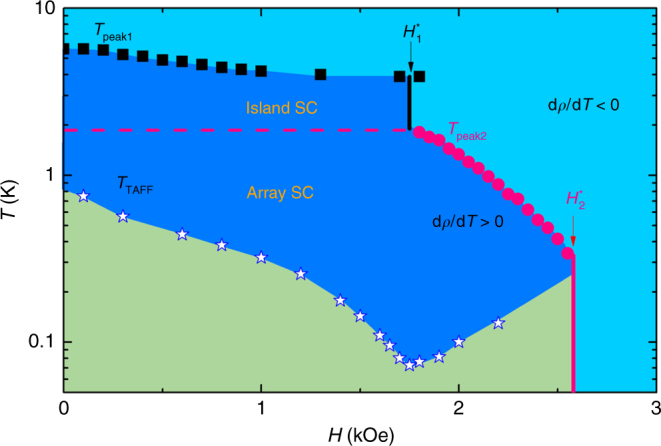
*H*–*T* phase diagram for the graphene-tin nanoislands array hybrid. The *T*_peak1 _(black square), *T*_peak2_ (red pink circle), HTCR (black solid line), and LTCR (red pink solid line) separate the superconductivity region (d*ρ/*d*T* > 0) and the weakly localizing metal region (d*ρ/*d*T* < 0). *T*_TAFF_ points, which correspond to the black arrows in the Arrhenius plot (Supplementary Figure 15), define the boundary between the thermal activation region and the quantum tunneling vortex region. Intra Island SC and Inter Island SC denote the intra-tin nanoisland superconductivity and the inter-tin nanoisland superconductivity, respectively

Despite the two quantum critical behaviors are well built at *H*_1_^*^ and *H*_2_^*^, respectively, the resistance scaling behavior at *H*_1_^*^ holds only down to 2 K instead of approaching to the lowest temperature, in contrast to the *H*_2_^*^. To account for the quantum critical behaviors of *H*_1_^*^ at lowest temperature, the *H*–*T* phase diagram has been constructed in Fig. 5. The *T*_peak_ data are extracted from the Fig. 3a with the black and pink arrows, which separate the superconductivity region (d*ρ/*d*T* > 0) and the weakly localizing metal region (d*ρ/*d*T* < 0). The scaling regions for the critical field *H*_1_^*^ and *H*_2_^*^ are also shown in Fig. 5. The scaling region corresponding to *H*_1_^*^ is about 2–3 K, which is slightly less than the tin bulk superconducting transition temperature *T*_c_ = 3.7 K and larger than the zero resistance temperature (∼1 K), while the scaling region corresponding to *H*_2_^*^ is around 0.05 ~ 0.2 K, which is approaching to the lowest temperature (50 mK) in present experiment. By analyzing the resistance data in the Arrhenius plot^4,5^ (Supplementary Figure 15), the blue stars *T*_TAFF_ inside the superconducting dome in Fig. 5 are obtained, which yield the boundary between the thermally activated flux flow region and the quantum tunneling region. It is striking to note that the boundary exhibits a V-shaped topology with the bottom located at the *H*_1_^*^ exactly. The *T*_TAFF_ line inside the superconducting phase generally reflects the crossover of vortex from the thermal activation to quantum tunneling mechanism^43^. In particular to the ultrathin superconducting disk-shaped nanoislands, the theory proposed that the magnetic field dependent *T*_TAFF_ line follows $T_{{\mathrm{TAFF}}} \propto \Phi - \Phi _0$ in the dissipative regime, where *Φ* and *Φ*_0_ are the magnetic flux and the quantum flux through a nanoisland, respectively^44^. Thus, for an ultrathin superconducting nanoisland, the *T*_TAFF_ is expected to reach the minimum at *Φ* = *Φ*_0_ and increases again once *Φ* is beyond *Φ*_0_, which yields a V-shape *T*_TAFF_ line as a function of *H*. Meanwhile, according to the previous report^14^ and our work, the critical field $H_1^ \ast \sim \Phi _0/L_{\mathrm{d}}^2$ at high temperature regime is the dephasing filed, which is determined by the nano-puddle size *L*_d_ and the quantum flux *Φ*_0_. Therefore, we assume the V-shape *T*_TAFF_ line should be related to the critical field $H_1^ \ast$. In addition, it is natural to imagine that tin islands start to become superconducting at high temperature regime, and extend to form a superconducting landscape consisting of superconducting islands and coupling regions as the temperature decreases. The dissipation scheme in our disordered system is believed to attribute to the vortices movement in the formed superconducting landscape, so it is plausible to observe the *T*_TAFF_ line of V-shape in a region where the inter-islands physics dominates at low temperature. As discussed above, it is concluded the two quantum critical behaviors correspond to the physics related to the intra-tin and inter-tin nanoisland length scales, suggesting the multiscale inhomogeneity can gives rise to multicritical behaviors. It may provide a new insight to the quantum many-body effects in the strongly correlated electronic systems like manganites and cuprates where the ground state has an inhomogeneous mixture of phases in nanoscale.

In summary, the 2D superconductor consisting of the superconducting tin nanoislands together with single crystalline monolayer graphene is synthesized on intrinsic Ge (110) wafer. This hybrid system can visually reproduce the superconducting puddles-2DEG system assumed at oxide interface, and the expected two QPTs induced by the magnetic field reappear as identified through FSS analysis on magnetoresistivity. The constructed *H*–*T* phase diagram shows that both QPTs correspond to the intra-tin nanoisland superconductivity and the inter-tin nanoisland superconductivity, respectively. This study provides an ideal visualized platform to investigate 2D superconducting QPT. In addition, the superconducting device arrays built on semiconductor germanium wafer sheds light on a great potential to enable the integration of superconducting devices with the existing semiconductor technology.

## Methods

### Graphene synthesis and characterization

Single crystalline wafer-scale monolayer graphene was synthesized on the intrinsic Ge (110) wafer by chemical vapor deposition (CVD). The Ge(110) wafer was loaded in a 4-inch CVD furnace. Afterwards, the chamber was evacuated to high vacuum. Then, the chamber was filled with the gas mixture of argon (Ar, 99.9999% purity) and hydrogen (H_2_, 99.9999% purity) to make the chamber to reach the atmospheric pressure. During the growth process, a certain proportion gas mixture of methane (CH_4_, 99.99% purity), hydrogen (H_2_, 99.9999% purity), argon (Ar, 99.9999% purity) was flowed in the chamber at 910 ^o^C for 300 min. Finally, the chamber was rapidly cooled down to room temperature under the flowing gas mixture of H_2_ and Ar.

The surface topography of graphene was measured by the tapping mode AFM (Multimode 8, Bruker) with RTESP AFM tips. Raman spectra measurement (HORIBA Jobin Yvon HR800) was carried out with the laser wavelength 514 nm and a spot size of 1 μm.

### Device fabrication and measurement

To avoid the possible contamination of the graphene surface by chemical polymer resist, we fabricated the device using the stencil mask which could maintain the intrinsic property of graphene. To exclude the possible shunt effect from the substrate, 4 inch intrinsic Ge (110) wafer (TaiCrystal, >50 ohm cm, 400 μm thickness) was chosen as the starting platform for the synthesis of graphene and the fabrication of Hall bar devices. The Hall bar device fabrication process started with the 10 nm Ti/100 nm Au electrode pattern, which was deposited utilizing the stencil mask to form ohmic contact with graphene. To define the graphene channel, the graphene except the channel graphene was etched using oxygen plasma aligned with the stencil mask. Finally, the 10 nm thick tin film was deposited on the sample by electron beam evaporation to complete the fabrication of hall bar device. *R*–*T* curve of Hall device was measured in physical property measurement system with He^3^-He^4^ dilution refrigerator (PPMS, Quantum design) with a base temperature of 50 mK.

### Data availability

The data that support the findings of this study are available from the corresponding author on reasonable request.

## Electronic supplementary material

Supplementary Information
