# Supplementary material for: Double quantum criticality in superconducting tin arrays-graphene hybrid
Source: Nat Commun. 2018 Jun 4;9:2159. doi: 10.1038/s41467-018-04606-w (PMC5986781; doi:10.1038/s41467-018-04606-w)
Supplement: Supplementary file 1 — Supplementary Information [file 41467_2018_4606_MOESM1_ESM.pdf]

Double quantum criticality in superconducting tin arrays-graphene hybrid

Sun et al.

Supplementary Note 1: Characterization of single crystalline graphene

Wafer scale single crystalline graphene has been successfully synthesized on 4 inch Ge wafer(Supplementary Figure 1a). The corresponding AFM images obtained from the selected regions show, except the randomly distributed wrinkles due to the negative thermal expansion coefficient of graphene, the flat graphene surface with extremely low roughness value is obtained (Supplementary Figure 1a). Raman spectra exhibits there is no appreciable D peak near 1350 cm^{-1}, indicating that the synthesized graphene possesses remarkable crystalline quality (Supplementary Figure 1b).

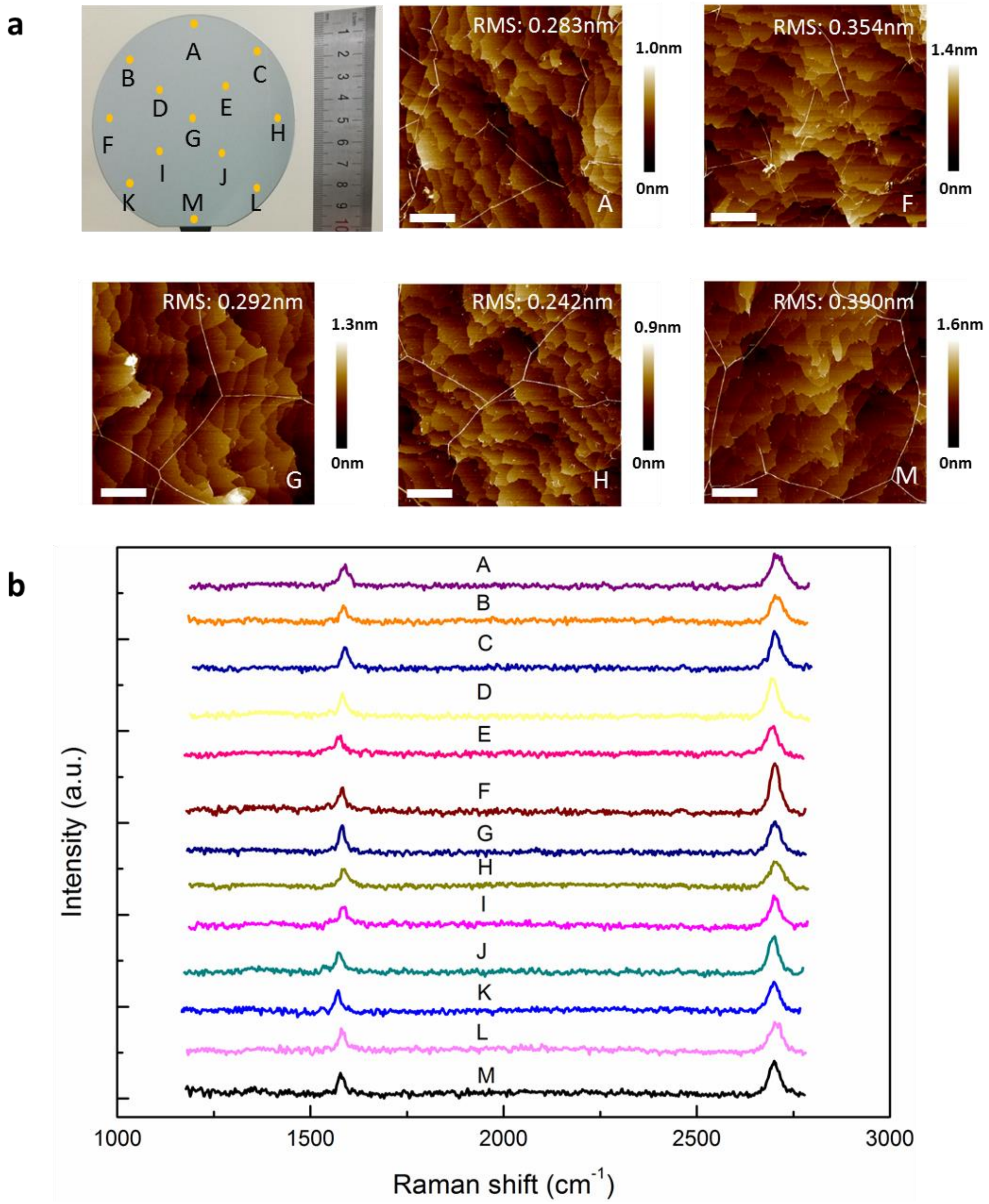

Supplementary Figure 1 | Wafer-scale single crystalline monolayer graphene grown on the germanium (110) wafer. **a**, Optical photography of 4 inch wafer-scale graphene and a set of AFM images obtained from the marked locations on the wafer. The scale bars for AFM images are 1 μm. **b**, The Raman spectra obtained from the selected locations on the wafer, as marked in a.

Supplementary Note 2: Device fabrication

The schematic illustration for the Hall bar device fabrication process was presented in Supplementary Figure 2. Firstly, single crystalline wafer-scale monolayer graphene was synthesized on the intrinsic Ge (110) wafer by chemical vapor deposition (CVD). Then, 10 nm Ti/100 nm Au electrode pattern was deposited utilizing the stencil mask to form ohmic contact with graphene. To define the graphene channel, the graphene except the channel graphene was etched using oxygen plasma aligned with the stencil mask. Finally, the 10 nm thick tin film was deposited on the sample by electron beam evaporation to complete the fabrication of hall bar device. Due to the low melting point of tin and the poor wettability of graphene, an array of self-assembled irregular tin nanoislands with lateral size of ~150 nm and interval of ~ 40 nm were distributed on graphene, as depicted in Supplementary Figure 3.

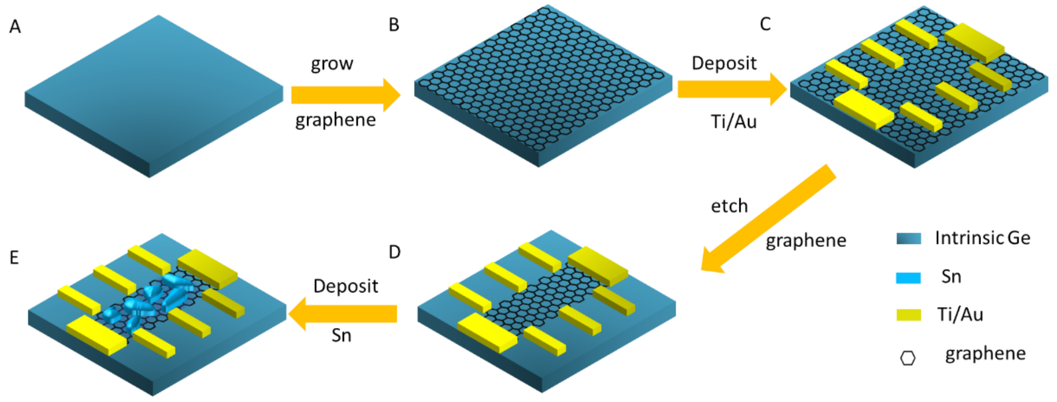

Supplementary Figure 2 | Sketch illustration of the Hall bar device fabrication. (A-B) Graphene grown on the intrinsic Ge(110) through CVD technique. (B-C) Au deposited on the graphene film through the electron beam evaporation using the stencil mask. (C-D) the removal of undesired graphene using the oxygen plasma for etching and the shadow mask as the protecting barrier. (D-E) Tin deposited on the graphene using the stencil mask to form the discontinuous nanoislands.

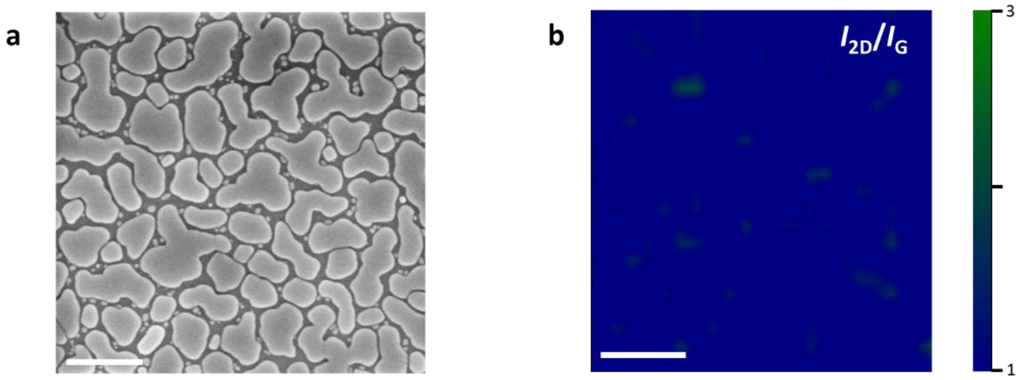

Supplementary Figure 3 | SEM images and 2D Raman mapping of the graphene-tin nanoislands array hybrid. (a) Scanning electron microscope (SEM) images of graphene-tin nanoislands hybrid. An array of self-assembled irregular tin nanoislands with discontinuity are formed on the graphene. The scale bar is 200 nm. (b) Raman mapping of the I_{2D}/I_G peak intensity ratio acquired from graphene-tin nanoislands hybrid. The uniform intensity ratio reveals the homogeneous graphene after the growth of tin nanoislands. The scale bar is 5 μm.

Supplementary Note 3: Transport properties

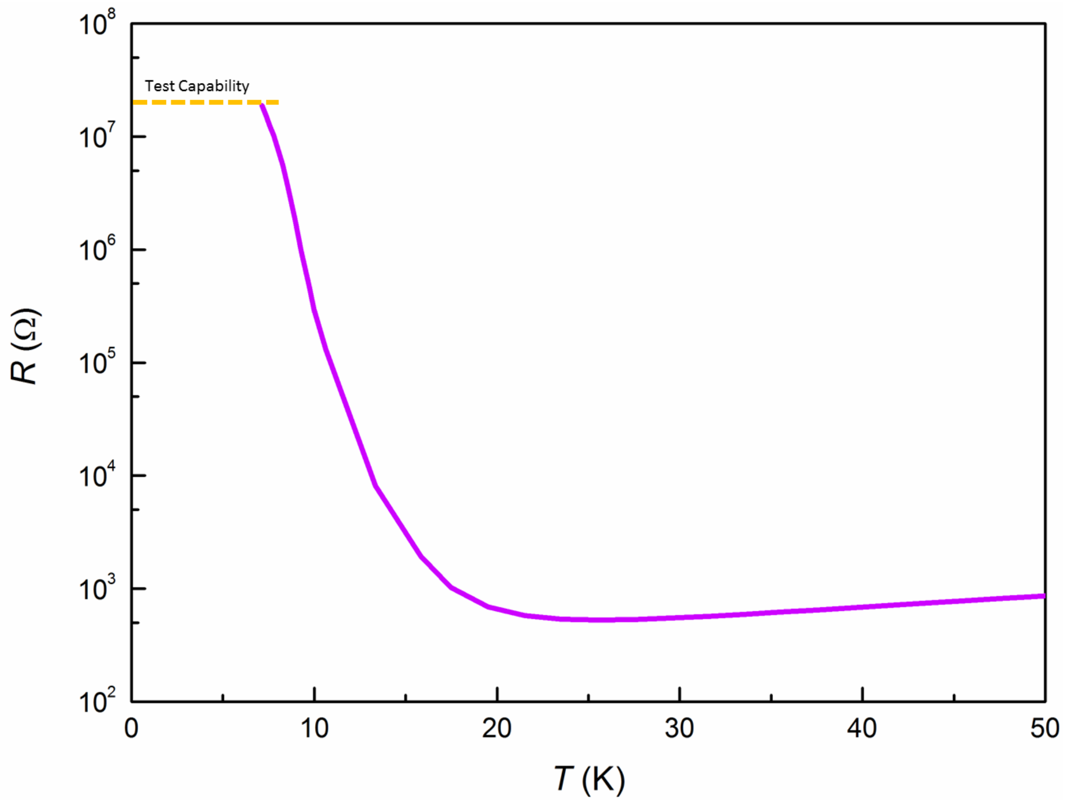

Supplementary Figure 4 | Insulating behavior of the intrinsic germanium (110). Temperature dependence of the resistance for intrinsic Ge(110). For comparison, the dimension of the measured region for the intrinsic Ge(110) is similar to that of the fabricated Hall bar device shown in the main text (Fig. 1). The intrinsic Ge(110) exhibits insulating behavior below 10 K.

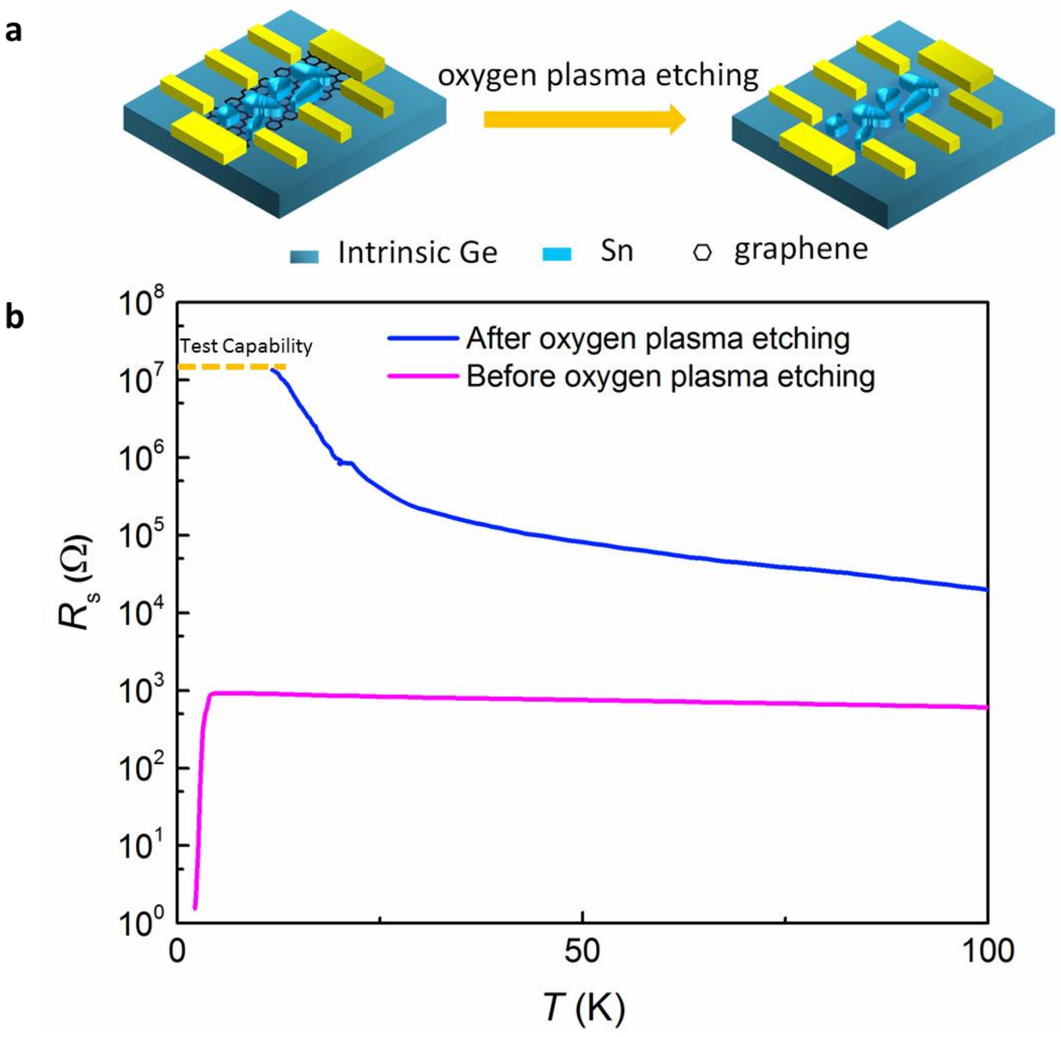

Supplementary Figure 5 | The comparison experiment to reveal the role of the graphene in forming the global superconductivity. **a**, The sketch illustration of the Hall bar device before and after the oxygen plasma etching. **b**, The *R*-*T* curves for the Hall bar devices before and after the oxygen plasma etching. It is shown that the Hall bar device becomes insulating after the removal of graphene between adjacent tin nanoislands by oxygen plasma etching, indicating that the graphene plays the essential role in forming the global superconductivity of the graphene-tin nanoislands array hybrid.

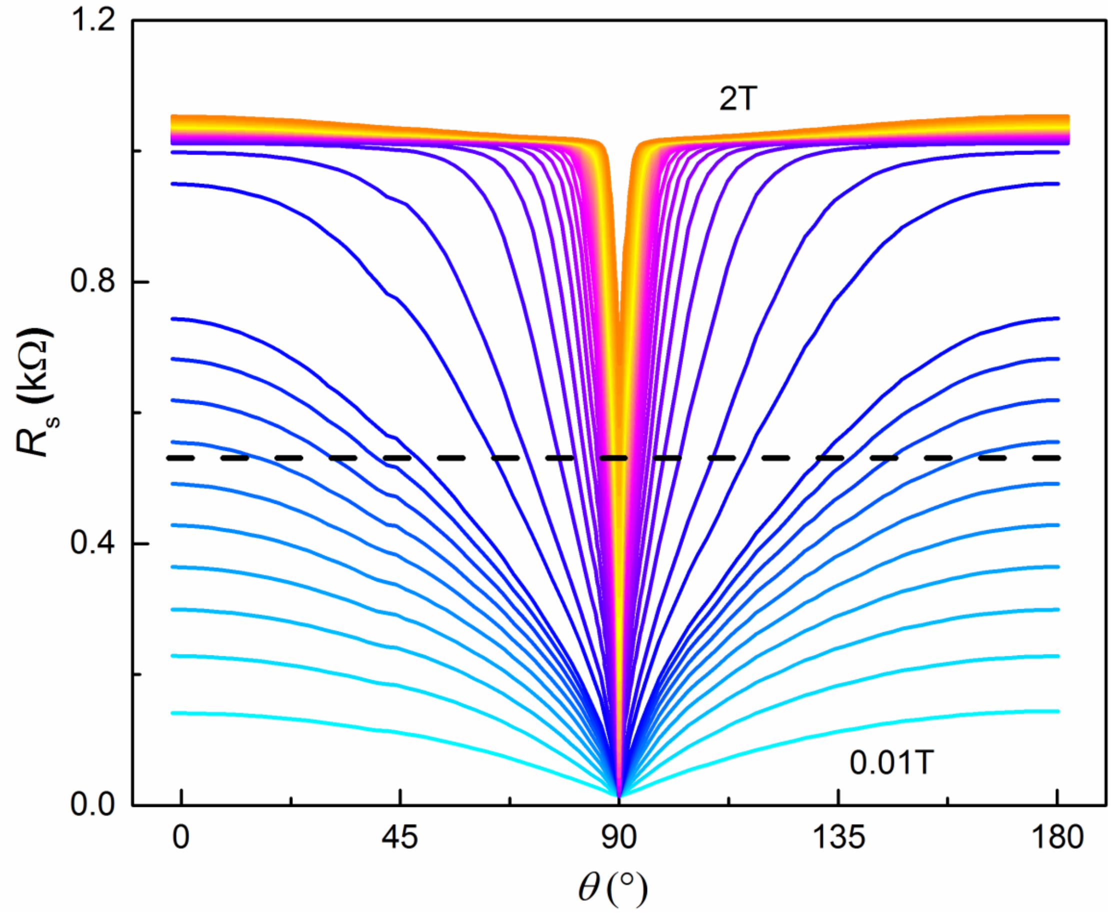

Supplementary Figure 6 | Angular dependence of the sheet resistance R_s for the graphene-tin nanoislands array hybrid at 2 K with various magnetic fields. The magnetic fields are 0.01 T~0.1 T (increment of field is 0.01 T), 0.15 T, 0.2 T~0.9 T (increment of field is 0.1 T), 1 T~2 T (increment of field is 0.05 T) from the bottom to the top. θ is the angle between the magnetic field and the c-axis of the sample surface. The black line indicates the 50% of the normal state sheet resistance.

Supplementary Note 4: Joule heating effect

The failure of cooling samples is quite common in the voltage-current (*V-I*) measurement for low dimension system at low temperature. To address this problem, we detected the temperature of our samples by the addition thermometer during the *V-I* measurement, as proposed by Huang, X. et al.[1] The experimental configuration is schematically drawn in Supplementary Figure 7(a), and the real image is displayed in Supplementary Figure 7(b). The thermometer chip is mounted on the sample using N-type grease and connected to the puck by the manganin wires. Due to the poor thermal conduction of manganin wires, the heat transport between the thermometer and the puck can be suppressed effectively. Prior to the temperature measurement, the thermometer has been calibrated, which exhibits the ratio of the resistance to the temperature $\Delta R/\Delta T$=140Ω/K at low temperature around 2 K, as shown in Supplementary Figure 8. For low temperature regime (2-4 K), it is found that the maximum change of the thermometer resistance is less than 10 Ω even the current reaches 2.5 mA (Supplementary Figure 9), suggesting the change of the sample temperature is less than 0.07 K (3% of 2 K) during the *V-I* measurement. Thus, we conclude that Joule heating is significantly minimized in our measurement and the non-linear *V-I* behaviors observed in Fig. 2c in the main text can be interpreted as the existence of Berezinskii-Kosterlitz-Thouless (BKT) transition.

In addition, to study whether such the non-linear *V-I* behavior exist in non-zero field, the *V-I* measurements were performed under different magnetic field at 2 K, as shown in Supplementary Figure 10. The non-linear *V-I* behavior exists only at low magnetic

fields, and disappears above 1000 Oe. For the magnet field larger than 1000 Oe, the *V*-*I* behavior becomes linear, corresponding to the ohmic characteristic. In our study, the finite size scaling analysis is always performed at the magnet field above 1000 Oe, i.e., the linear *V*-*I* region (ohmic region), therefore, the scaling resistance we obtained is reliable and not affected by the Joule heating.

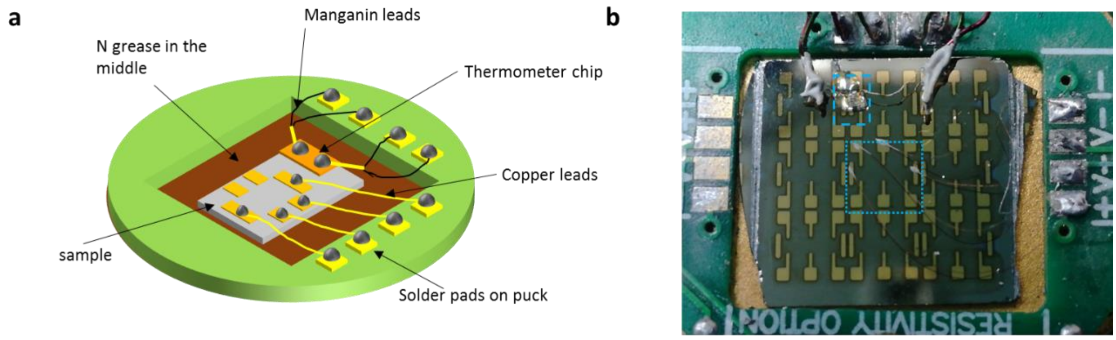

Supplementary Figure 7 | The experimental configuration for clarifying the Joule heating effect during the voltage-current measurements. a, The sketch illustration of the measurement configuration. **b**, The image of the measurement configuration. The thermometer is marked by the dashed square, and the sample is marked by the dotted square.

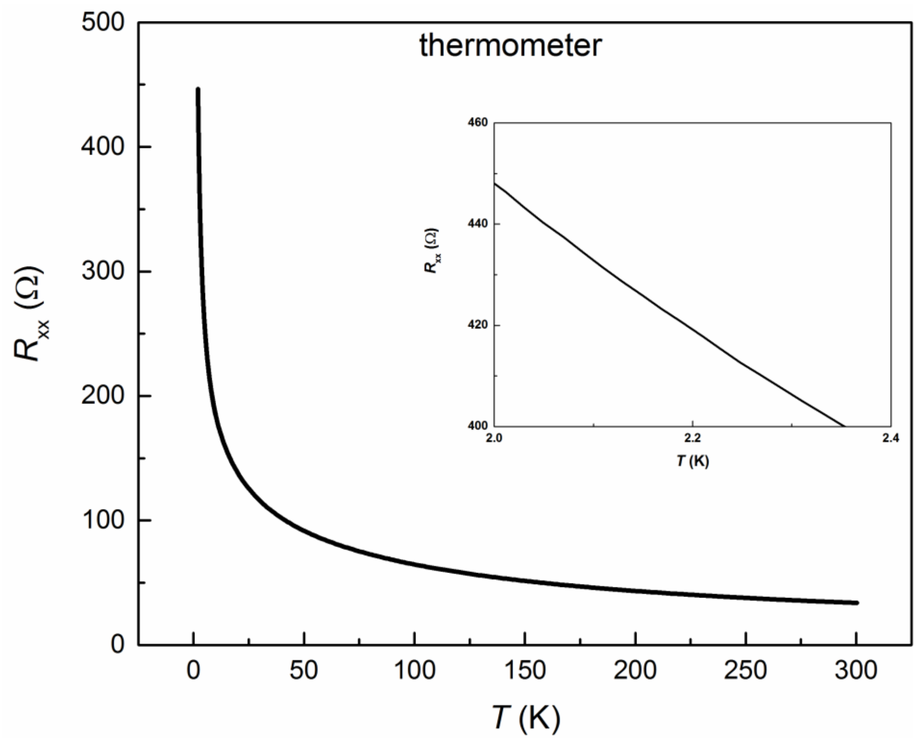

Supplementary Figure 8 | Resistance changes in the thermometer as a function of the measuring temperature. The inset shows the resistance changes at the low temperature around 2 K.

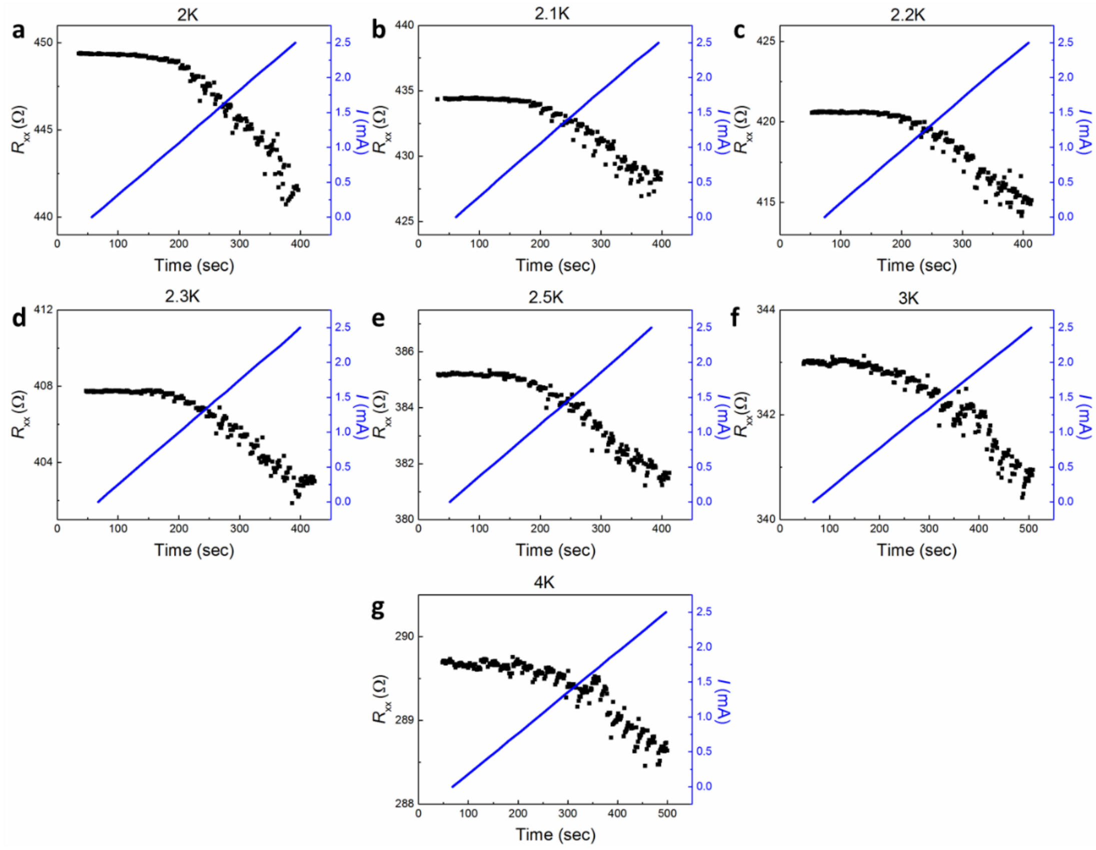

Supplementary Figure 9 | The resistance change in the thermometer for different applied current at various temperatures from 2 to 4 K.

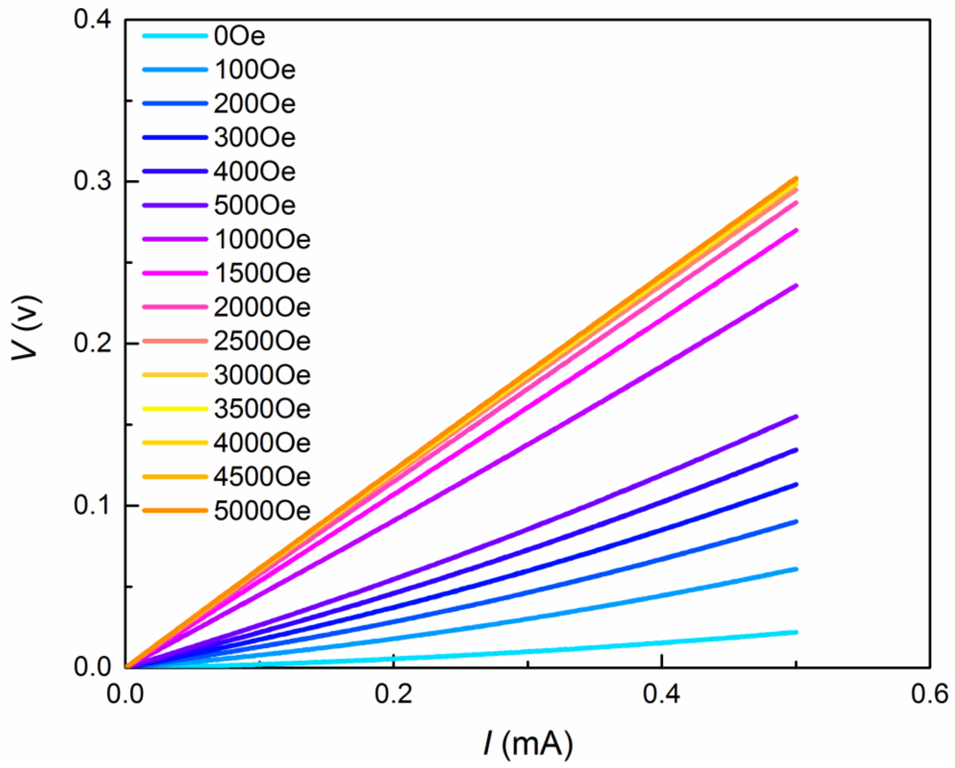

Supplementary Figure 10 | The measured voltage-current curves at 2 K under different magnetic field. Non-linear *V*-*I* behaviors exist at low magnetic fields below 1000 Oe.

Supplementary Notes 5: The sheet resistances under magnetic field

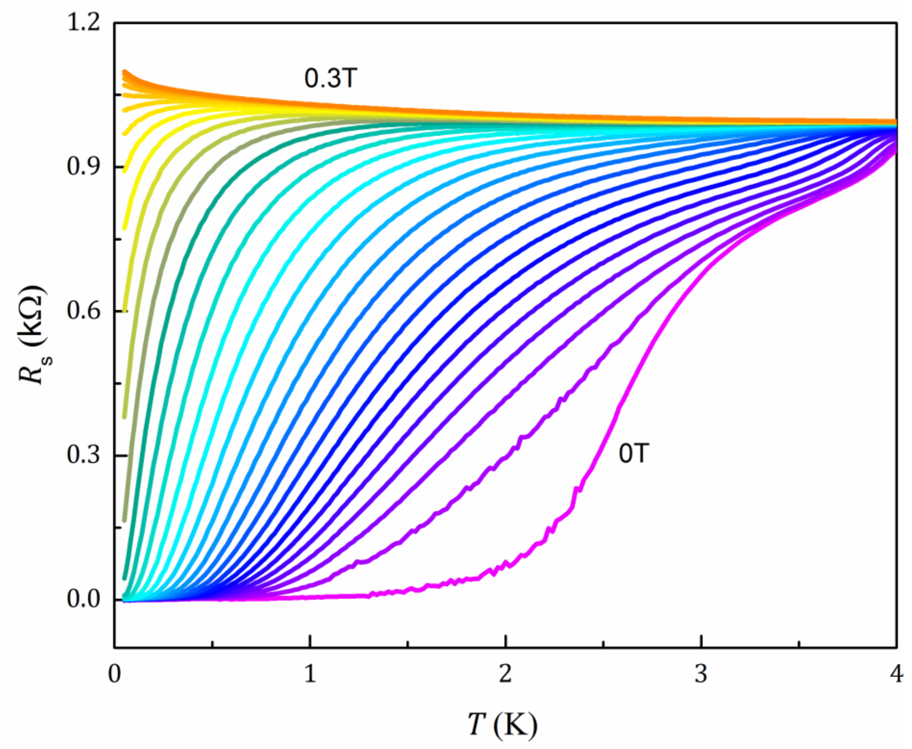

Supplementary Figure 11 | Sheet resistance R_s as a function of the temperature for the graphene-tin nanoislands array hybrid. The temperature dependence of the sheet resistance at different perpendicular magnetic field varying from 0 to 0.3 T. The increment of the field is 0.01 T.

Supplementary Notes 6: The data collapsing with different *zv* value

We show the finite size scaling with different *zv* value at the high temperature region (2 K~2.5 K) and low temperature region (0.05 K~0.2 K), respectively, as shown in Supplementary Figure 12, Supplementary Figure 13. For high temperature region, the best collapsing has been achieved at *zv* = 0.63 when the *zv* value changes from 0.1 to 1. For low temperature region, the perfect collapsing is reached at *zv* = 3.85 as the *zv* value varies between 1 and 5. Therefore, the data collapsing really correlates with the value of *zv*, and the *zv* values reported in the main text are reliable and accurate.

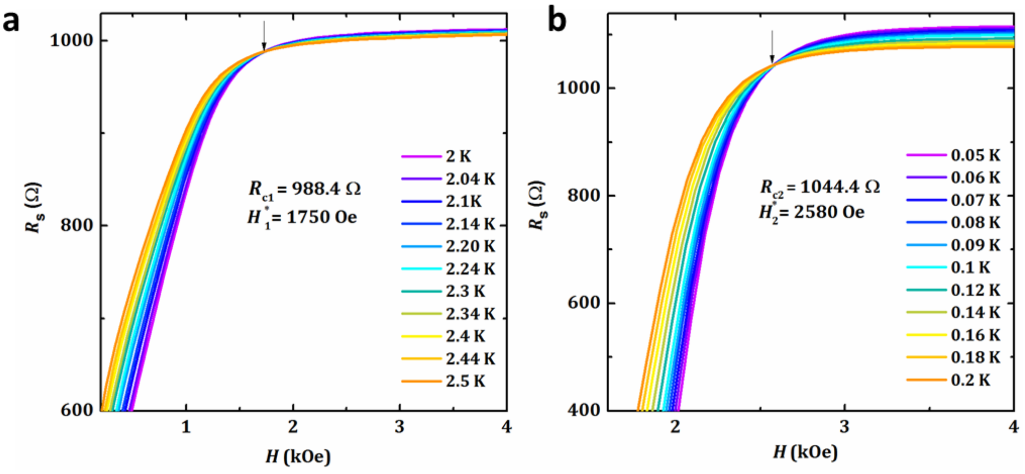

Supplementary Figure 12 | Sheet resistance R_s as a function of the magnetic field *H* for different temperature. (a) 2 to 2.5 K; (b) 0.05 to 0.2 K. The data include larger sheet resistance range and larger magnetic field range. The data well converge at the critical magnetic field H_1*and H_2*, and the critical resistance R_{c1} and R_{c2}, respectively.

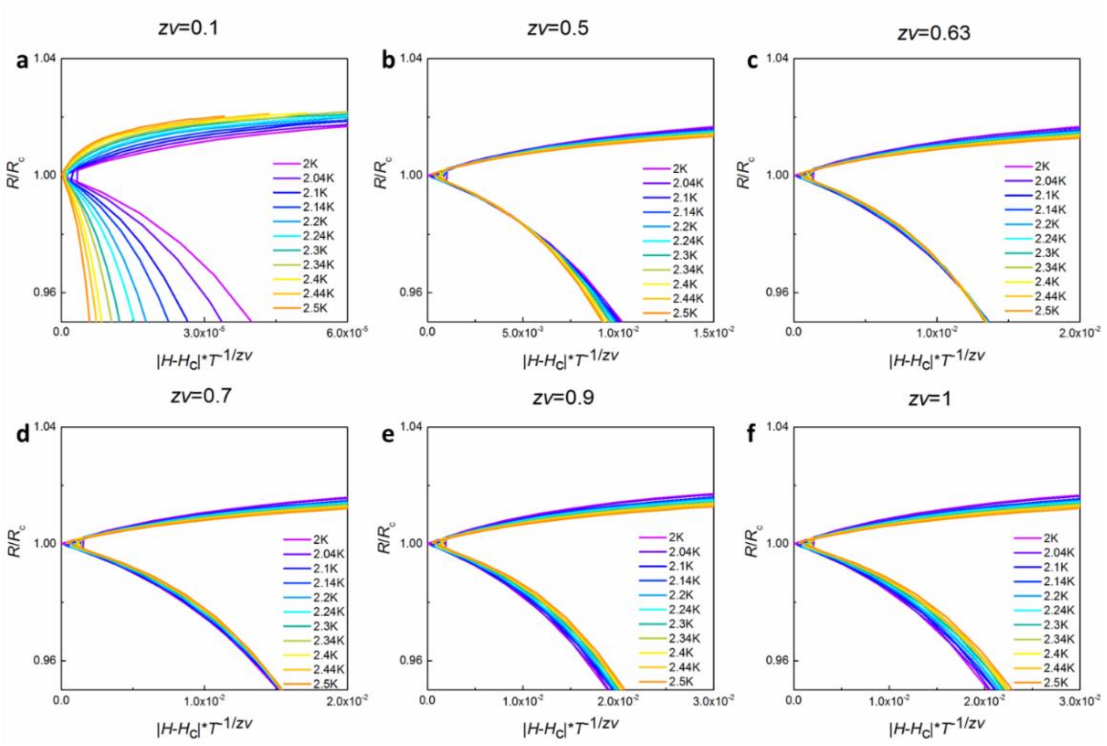

Supplementary Figure 13 | The finite size scaling process using different *zv* value at the temperature ranging from 2 to 2.5 K. The best data collapsing has be achieved at *zv* = 0.63 as reported in the main text.

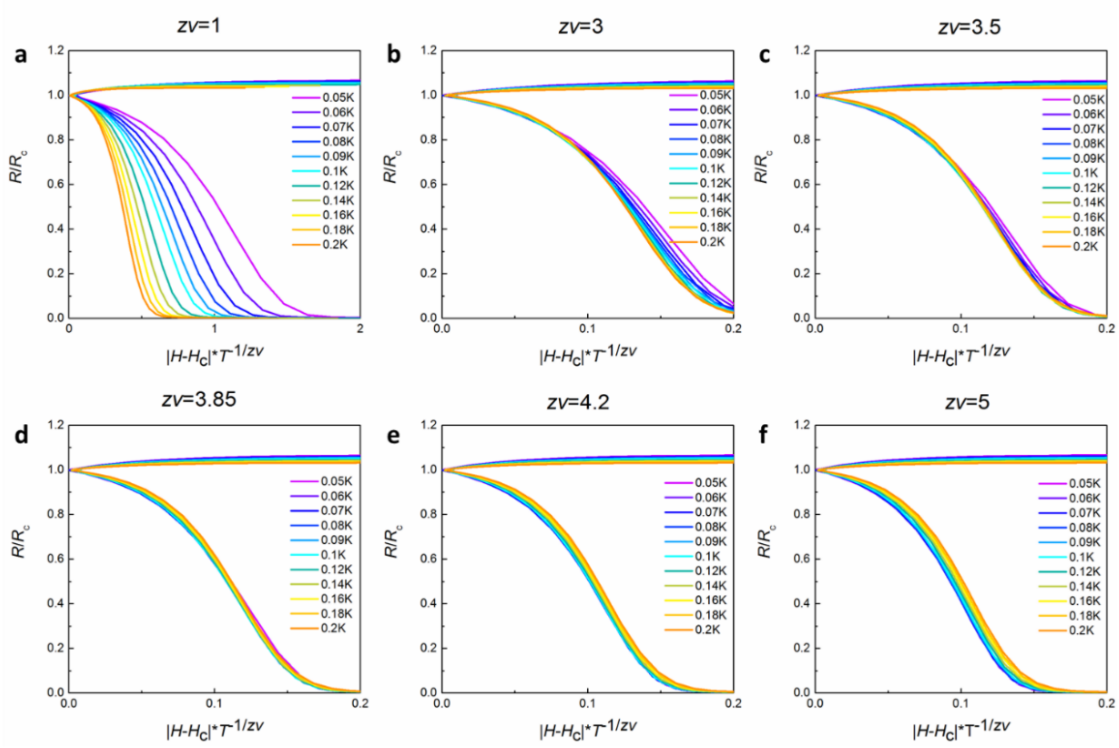

Supplementary Figure 14 | The finite size scaling process using different *zv* value at the temperature ranging from 0.05 to 0.2 K. The best data collapsing has be achieved at *zv* = 3.85 as reported in the main text.

Supplementary Notes 7: Arrhenius plot

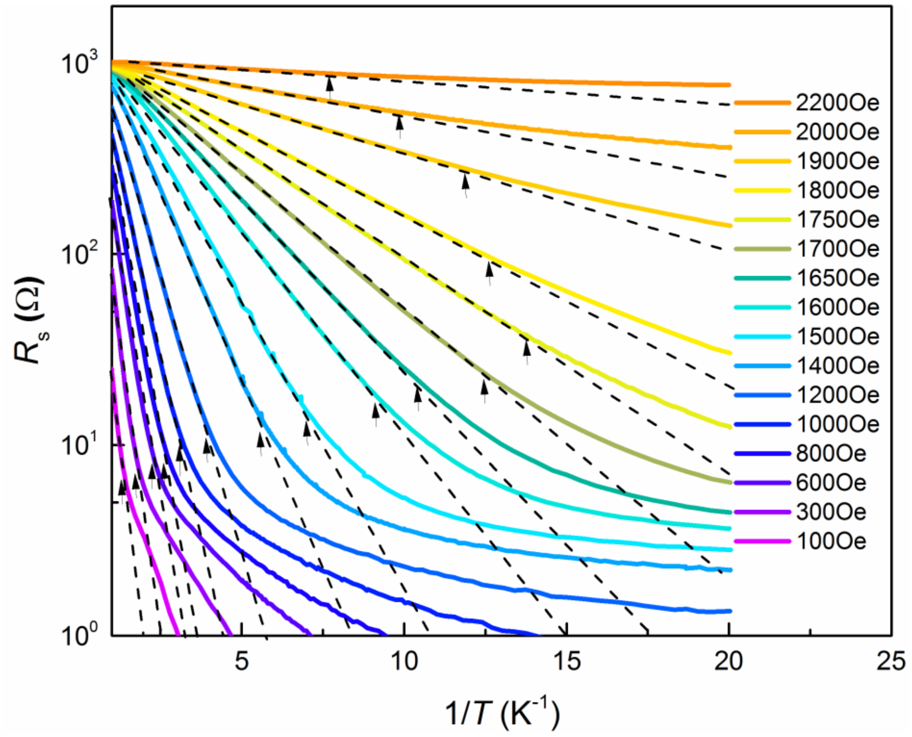

Supplementary Figure 15 | Arrhenius plot of the sheet resistance R_s for the graphene-tin nanoislands array hybrid at different perpendicular magnetic field. The black dashed lines indicate the thermally activated behavior depicted by the formula: $R_s = R^{'} \exp(-U(H)/k_B T)$ where $U(H)$ and k_B denote the activation energy and the Boltzmann's constant, respectively. The black arrows indicate the ends of the thermally activated behaviors, which correspond to the blue stars T_{TAFF} in Fig. 5 of the main text.

Supplementary References

1 Huang, X. *et al.* Universality and unconventional enhancement of flux-flow resistivity in Ba (Fe 1− x Co x) 2 As 2. *Physical Review B* **95**, 184513 (2017).
